# A 1-week extension of a ketogenic diet provides a further decrease in myocardial ^18^F-FDG uptake and a high detectability of myocarditis with FDG-PET

**DOI:** 10.1007/s12350-018-1404-7

**Published:** 2018-08-20

**Authors:** Alexandra Clément, Henri Boutley, Sylvain Poussier, Julien Pierson, Mickael Lhuillier, Allan Kolodziej, Jean-Luc Olivier, Gilles Karcher, Pierre-Yves Marie, Fatiha Maskali

**Affiliations:** 1 Nancyclotep, Molecular and Experimental Imaging Platform, Brabois Hospital, 54505 Vandœuvre-lès-Nancy, France; 2 Department of Biochemistry and Molecular Biology, CHRU-Nancy, University of Lorraine, 54000 Nancy, France; 3 Department of Nuclear Medicine, CHRU-Nancy, University of Lorraine, 54000 Nancy, France; 4 University of Lorraine, INSERM, UMR 1116, 54000 Nancy, France

**Keywords:** ^18^F-fluorodeoxyglucose, positron emission tomography, fasting, low-carbohydrate diet, myocarditis

## Abstract

**Background:**

Short periods of fasting and/or low-carbohydrate diet have been proven beneficial for decreasing the myocardial uptake of ^18^F-fluorodeoxyglucose (^18^F-FDG) and enhancing the detection of inflammatory heart diseases by ^18^F-FDG positron emission tomography (PET). This study aimed at determining whether this benefit is increased when a low-carbohydrate ketogenic diet is prolonged up to 7 days.

**Methods:**

Wistar rats underwent serial ^18^F-FDG-PET imaging after an 18-hour fasting period and after 2, 4 and 7 days of a ketogenic diet (3% carbohydrate) and they were compared to rats submitted to the same protocol but with normal diet (44% carbohydrate). The ^18^F-FDG-PET/ketogenic protocol was also applied in rats with immune myocarditis (injection of porcine cardiac myosin).

**Results:**

The 7-day ketogenic diet was associated with (1) a sustained increase in circulating ketone bodies at an equivalent level to that reached after 18-hour fasting, (2) a gradual decrease in ^18^F-FDG uptake within normal myocardium reaching a lower level compared to fasting at the 7th day (myocardium-to-blood ratios: 1.68 ± 1.02 vs 3.25 ± 1.40, *P* < .05) and (3) a high ^18^F-FDG-PET detectability of myocarditis areas.

**Conclusion:**

One-week extension of a ketogenic diet provides a further decrease in the ^18^F-FDG uptake of normal myocardium and a high detectability of inflammatory areas.

**Electronic supplementary material:**

The online version of this article (10.1007/s12350-018-1404-7) contains supplementary material, which is available to authorized users.

## Introduction

A fasting period of at least 12 hours,^1^-^4^ as well as low-carbohydrate diet,^5^-^12^ have been proven beneficial for decreasing the normal myocardial uptake of ^18^F-fluorodeoxyglucose (^18^F-FDG) and for enhancing the detectability of inflammatory and/or infectious heart diseases by ^18^F-FDG positron emission tomography (^18^F-FDG-PET). Unfortunately, none of these previously reported protocols enable the complete and consistent suppression of the cardiac uptake of ^18^F-FDG.

This study was aimed at determining whether a drastic ketogenic diet provides a further decrease in physiological myocardial ^18^F-FDG uptake and a high detectability of myocarditis by ^18^F-FDG-PET in rats, when this diet is prolonged up to 1 week, as compared with a standard 18-hour fasting conditioning. Such diet can be prescribed at a much longer term than fasting,^13^-^15^ leading to metabolic changes that are known to progressively suppress the metabolic use of glucose, even in the brain, within at least a 3- to 5-day period.^15^-^18^

## Methods

### Study Design and Experimental Groups

All protocols were approved by the Lorraine Committee No. 68 according to Guidelines of Animal Care and Use (*APAFIS # 1755-201509151127522v1*).

Twelve adult male Wistar rats underwent cardiac ^18^F-FDG-PET after an 18-hour fasting period (day-0) and subsequently after 2, 4 and 7 days of a ketogenic diet^19^,^20^ (3% carbohydrate, 73% fat, 15% protein, 0% fiber, and 9% vitamins and minerals; ketocal^®^, SDS, France). This human pharmacological ketogenic product was processed in biscuits by adding water, as already described in previous rat experiments.^19^,^20^ The experimental animals were compared to a control group of 7 rats submitted to the same protocol but with a normal diet (44% carbohydrate, 6% fat, 19% protein, 18% fiber, and 13% vitamins and minerals; Envigo, Gannat, France). Food and water were given ad libitum in both ketogenic and control groups and each rat was weighted daily, at a fixed time, all along the experimental protocol and with a dedicated small-animal weighing balance (Mettler Toledo, DeltaRange-PR5002, France).

Blood venous samples of approximately 1 mL were collected in EDTA tubes from the tail vain just before each ^18^F-FDG injection and thereafter, blood was centrifuged at 3000 g for 15 minutes, the plasma being subsequently frozen at − 80 °C for further ketones bodies measurements (β-Hydroxybutyrate Assay Kit MAK041, Sigma, Saint-Quentin-Fallavier, France). The kit is designed to produce a compound whose colorimetric intensity, determined at a wavelength of 450 nm, is proportional to the concentration of β-hydroxybutyrate.

Animals were sacrificed after the last PET recording for subsequent histological and autohistoradiographic studies.

The same PET/ketogenic diet protocol was also applied to five rats with an immune myocarditis, starting 6 weeks after the subcutaneous injection of an emulsion of 1 mg (10 mg·mL^−1^) of porcine cardiac myosin (Sigma, Saint-Quentin-Fallavier, France).^21^

Approximately 74 MBq of ^18^F-FDG were injected in the caudal vein under brief anesthesia (1.5%-2.5% isoflurane inhalation), 60 minutes prior to initiating PET acquisition. This acquisition was obtained under the same isoflurane anesthesia with a dedicated small-animal PET system (Inveon, Siemens, Knoxville, Tennessee, USA), as previously described elsewhere.^22^-^24^ The animals were positioned in prone position on a heating pad with a recording time of 30 minutes for ^18^F emission and 10 minutes for ^57^Co transmission.

Images were reconstructed in 16 cardiac intervals, and the corresponding 16 sinograms were reconstructed with a 3D OSEM algorithm with attenuation and scatter correction according to the following parameters: 4 iterations, 128 × 128 matrix, 2.0 zoom, and 0.8 mm thickness, leading to a voxel size of 0.8 × 0.4 × 0.4 mm. The study groups were compared according to their myocardial/blood activity ratio (M/B), an index of myocardial ^18^F-FDG uptake.^5^,^22^-^24^ As previously described,^22^ this ratio was estimated on a single mid-ventricular end-diastolic short-axis slice with mean myocardial counts being determined using a half-moon-shaped region of interest and mean blood counts, on a sphere of 1.5 mm in diameter positioned at the center of the LV cavity.^22^

### Histological Section Analysis

At the end of the study, animals were sacrificed by sodium pentobarbital overdose (180 mg·kg^−1^) and their hearts were excised and snap frozen in isopentane cooled with liquid nitrogen. Contiguous 8 μm sections were obtained with a cryostat at − 22 °C for autohistoradiography and histological staining, respectively.

Distribution of ^18^F-FDG activity was recorded with an autohistoradiography system dedicated to the detection of electrons and positrons (µImager™, Biospace, France).^22^ For Hematoxylin-Eosin-Safran (HES) staining, the sections were fixed in 95% ethanol, stained with hematoxylin for 1 minute, eosin and safran for 30 seconds each, before being dehydrated in ethanol 100% and xylene. For the Masson trichrome staining, the sections were fixed by immersion in Bouin solution for 15 minutes and picric acid for 5 minutes. The nuclei were stained with Weigert hematoxylin for 10 minutes, cytoplasm and smooth muscle with Biebrich solution and collagen fibers by immersion for 5 minutes in aniline blue.

For further immunohistology analyses, adjacent 5 µm sections were fixed with paraformaldehyde 4% (VWR, Fontenay-sous-Bois, France), incubated with a rabbit polyclonal antibody to determine macrophage infiltrates (anti-Vimentin antibody; 1:500; Dako, Les Ulis, France).

### Statistical Analyses

All data are expressed as mean ± standard deviation (SD). Statistical analyses were performed using the SPSS Statistics Software package v. 20 (IBM, NY, USA). Comparisons of quantitative variables were performed with ANOVA-test, after verifying for distribution normality. *P* values < .05 were considered as statistically significant.

## Results

**Figure 1:**
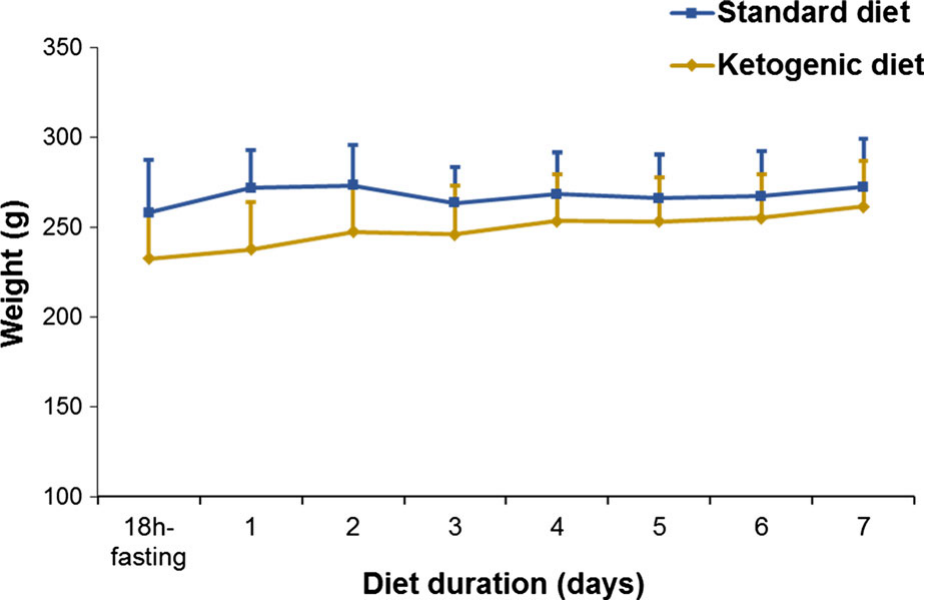
Comparison of mean body weight between rats fed with the ketogenic diet and control rats fed with a normal diet, throughout the experimental period. No significant differences in mean body weight were observed

**Figure 2:**
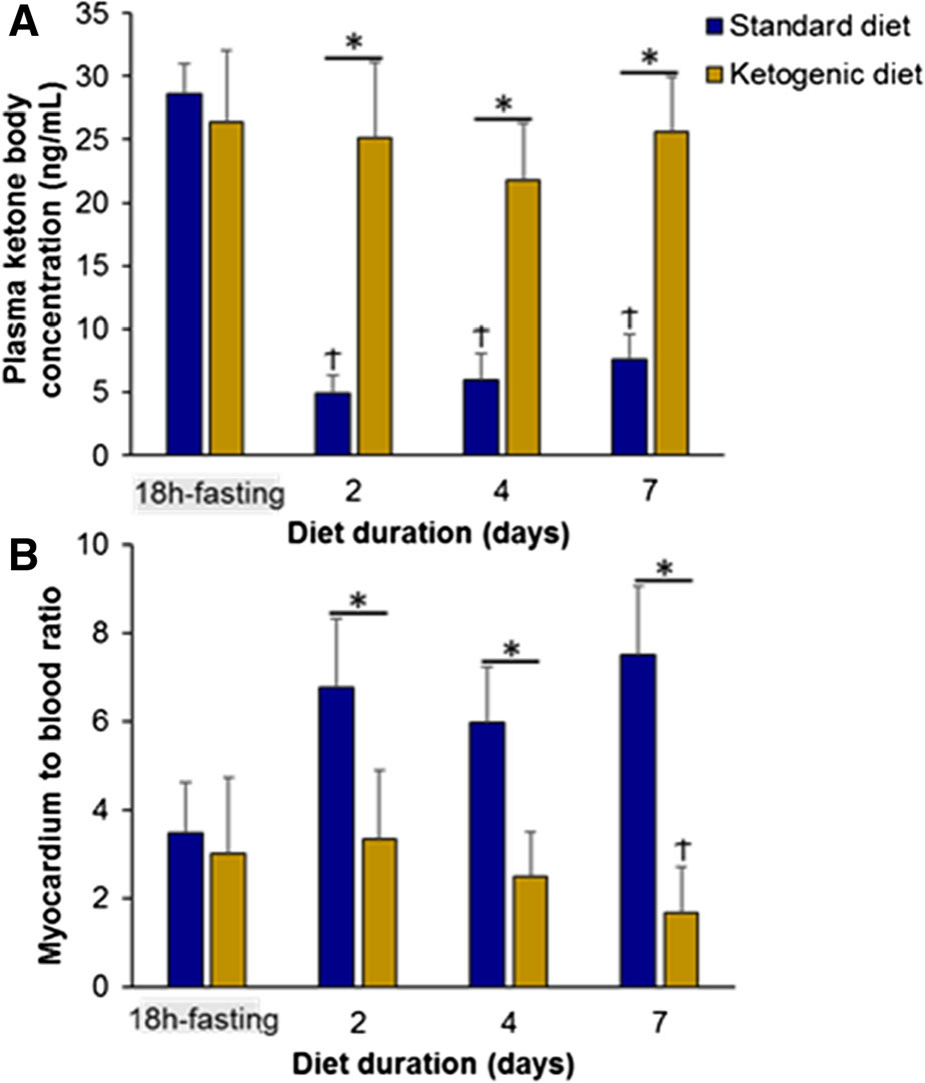
(**A**) Plasma concentrations of ketone body determined after the initial 18-hour fasting period and thereafter, throughout the standard and ketogenic diet periods (*P < .05 for two-group comparisons and ^†^*P* < .05 paired comparisons with the 18-hour fasting period). (**B**) Myocardium-to-blood activity ratio determined in vivo on [18F]-FDG-PET images after the initial 18-hour fasting period and thereafter, throughout the standard and ketogenic diet periods (*P < .05 for two-group comparisons and ^†^*P* < .05 paired comparisons with the 18-hour fasting period)

As detailed in Figure 1, mean body weight was equivalent between rats fed with the ketogenic diet and control rats throughout the experimental period (at the 7th day: 261 ± 25 g vs 272 ± 30 g, NS). By contrast, a sustained increase in the level of circulating ketone bodies was documented throughout the ketogenic diet period, contrary to that documented in the standard diet control group during the same period (Figure 2A). The level achieved at the 7th day of ketogenic diet was as high as that observed after the initial 18-hour fasting period (Figure 2A).

**Figure 3:**
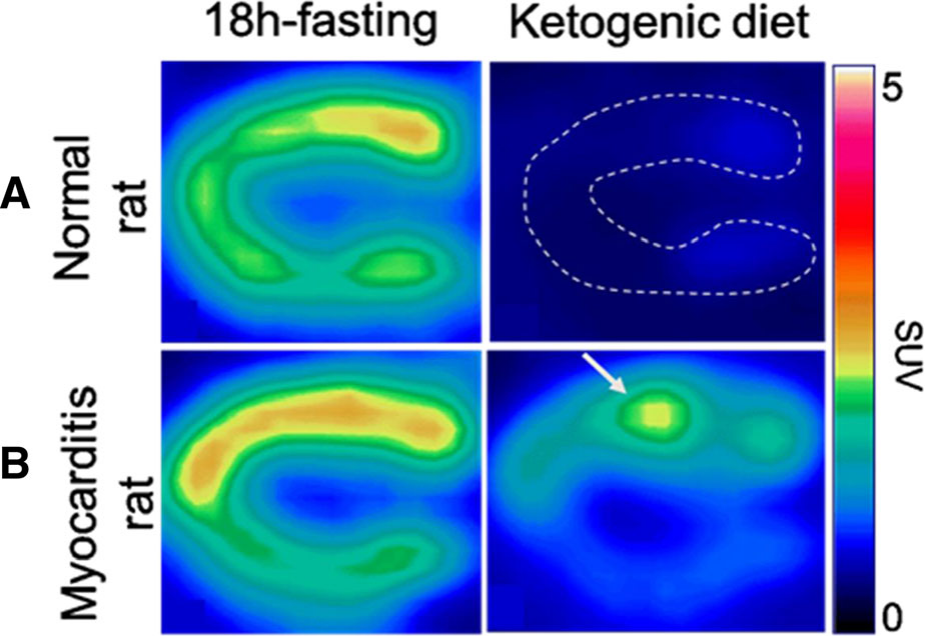
Representative images of the left ventricle obtained with [18F]-FDG-PET in a vertical long-axis orientation in a myocarditis rat (**B**) and in a normal rat (**A**) both following the initial 18-hour fasting period and at the end of the 7-day ketogenic diet. Note that the level of [18F]-FDG activity within normal myocardium is much lower after the ketogenic diet than after the 18-hour fasting period, allowing an easy delineation of a myocarditis anterior focus. Demonstrative cine-loop images of the same rats are available in a supplemental file

In addition, ^18^F-FDG myocardial uptake, assessed by means of a myocardial-to-blood activity ratio (Figure 2B), exhibited a gradual decline throughout the ketogenic diet period, reaching a lower level at the 7th day than that documented with the initial 18-hour fasting period (1.68 ± 1.02 vs 3.25 ± 1.40, *P* < .05). Representative ^18^F-FDG-PET images are displayed in Figure 3A.

As shown in Figure 3B and especially on the cine-loop images available in a supplemental (online) file, the delineation of myocarditis areas by ^18^F-FDG-PET was clearly evident at the 7th day of the ketogenic diet owing to a high contrast with normal myocardium.

**Figure 4:**
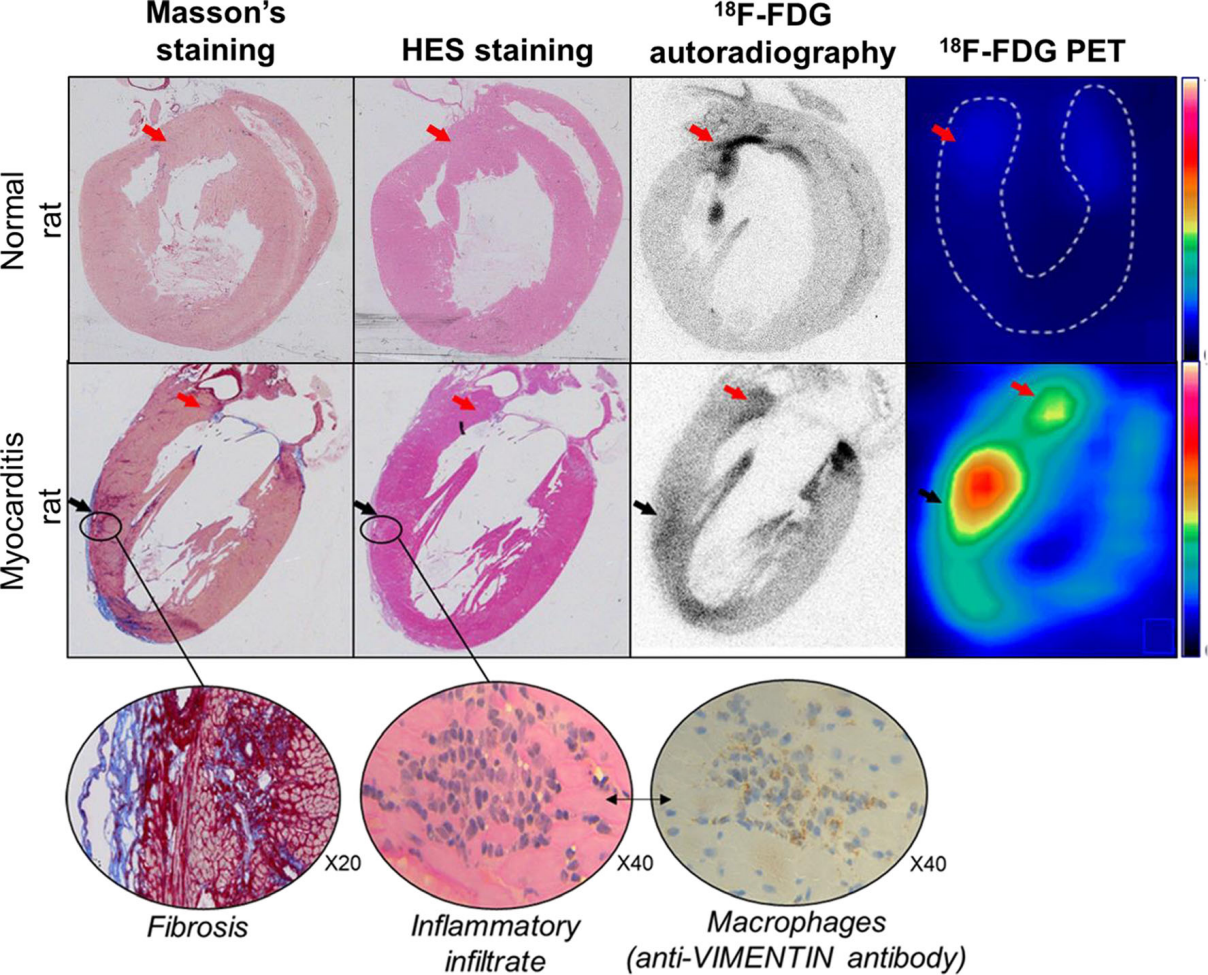
Images of the left ventricle obtained through a vertical long-axis orientation in the normal rat and myocarditis rat and at the end of the 7 days of the ketogenic diet (1) with the distribution of [18F]-FDG activity obtained in vivo with the PET camera and thereafter, ex vivo at autohistoradiography, both showing the anterior and apical myocarditis areas (black arrows), as well as small areas of increased [18F]-FDG uptake in contact with the mitral annulus (red arrows), and (2) with the colocalization of fibrosis (red color with Masson’s staining) and inflammatory infiltrates (blue color with HES staining) and macrophages (brown color with antibody anti-VIMENTIN staining) on contiguous histological slices

Moreover, as illustrated in Figure 4, the histological sections from myocarditis rats demonstrated that the myocardial areas showing an increase in ^18^F-FDG uptake mostly corresponded to a sub-acute myocarditis, with evidence of an increased fibrosis and of an inflammatory infiltrate at the corresponding sites. The cardiac uptake of ^18^F-FDG was additionally found somewhat higher around the mitral annulus in the ketogenic rats with or without myocarditis (Figures 3, 4).

## Discussion

This study shows that, when compared with a rather long fasting period of almost 18 hours,^8^,^25^ a 1-week extension of a drastic ketogenic diet provides a further decrease in myocardial ^18^F-FDG uptake and consequently, a high detectability of myocarditis by ^18^F-FDG-PET.

Current recommendations for an overnight fast after a last meal with low-carbohydrate intake are likely to prove inadequate in a significant proportion of patients for whom detection of inflammatory and/or infectious heart diseases is attempted by ^18^F-FDG-PET.^1^,^5^-^7^ More prolonged periods of fasting up to 18 hours could potentially constitute a more efficient method to switch the myocardial metabolism to a preferential use of ketone bodies and free fatty acids, thereby leading to a decrease in the cardiac uptake of glucose and ^18^F-FDG.^1^-^3^,^7^,^26^ Fasting duration is a key point in this setting, with a marked impact on the ability of ^18^F-FDG-PET to diagnose inflammatory heart diseases, as shown in a recent meta-analysis performed in cardiac sarcoidosis patients.^7^ Unfortunately, prolonged fasting of more than 12 or 18 hours may still provide a significant proportion of suboptimal results 1-3,7,26 and is not easily applied in certain severely ill patients with suspected endocarditis or myocarditis.

Low-carbohydrate diet protocols constitute a much more secure alternative for decreasing the myocardial uptake of ^18^F-FDG.

Such protocols have previously been shown to be well tolerated, even when prolonged several weeks or months in various diseases, including with an established efficacy in pediatric pharmaco-resistant epilepsy.^13^-^15^ Ketogenic diet may also be prescribed in diabetic patients without significant risk and at the condition of adapting the antidiabetic treatment to the improvement in blood glucose levels and to the reduction of the need for insulin, which are currently induced by such diets.^30^ In addition, low-carbohydrate diets have shown a significant albeit variable effectiveness for decreasing myocardial ^18^F-FDG uptake in a number of ^18^F-FDG-PET studies conducted in humans 4,8-10 or animals.^11^,^12^ The variability of this effectiveness is likely attributable to differences in diet protocols and particularly in the duration and in the degree of carbohydrate reduction. Sustained periods of dietary carbohydrate restriction lasting several weeks in animals^12^ or at least several days in humans^8^ have been shown to provide a relatively stable and marked reduction in cardiac ^18^F-FDG uptake. By contrast, the impact of uncontrolled short diet periods lasting no more than 24 hours did not enhance the results provided by fasting in a large previous meta-analysis.^7^ These observations are in agreement with the previous knowledge that drastic carbohydrate reduction, prolonged at least 3-5 days, are required to definitely enter into a state of ketosis.^15^ After this delay-time, the glucose reserves become insufficient, both for normal fat oxidation via the supply of oxaloacetate in the Krebs cycle and for the supply of glucose, even in the central nervous system.^15^,^16^

The present experimental study is the first in which the impact of such a drastic diet, leading to an increase in circulating ketone bodies at a very high level and equivalent to that reached by a prolonged 18-hour fasting period, could be monitored by serial ^18^F-FDG-PET during a 7-day period. In these conditions, cardiac ^18^F-FDG uptake exhibited a gradual decrease over time up to a very low level on the 7th day, in agreement with the progressive development of the ketosis state, as stated above.

This prolonged diet was additionally found i) to be well tolerated, as evidenced by the absence of any significant loss in body weight, and ii) to provide a normal cardiac ^18^F-FDG uptake more than twofold lower on the 7th day of the diet than that achieved with the 18-hours fasting. In these conditions, areas of sub-acute myocarditis could be easily delineated because of a high contrast from normal myocardial areas, as evidenced by the comprehensive analysis of PET images and histopathological sections (Figure 4). Accordingly, areas of high ^18^F-FDG activity in these sections were shown to be associated with increased fibrosis, as well as with a high density of inflammatory infiltrate and macrophages. The anti-vimentin antibodies used in this study are likely to mainly label macrophages in this particular setting of myocarditis, even if it must be recognized that this antibody is not highly specific for this purpose. It should be pointed out that the areas of increased fibrosis were not only those corresponding to the evolving sub-acute myocarditis, but also those physiologically documented at the LV base, in the vicinity of the mitral annulus. This was associated with a ring-like uptake at the LV base, a pattern previously documented by ^18^F-FDG-PET in normal healthy volunteers after low-carbohydrate and fasting diets.^9^,^27^ This may be explained by the fact that these fibrotic regions are rich in fibroblasts, expressing GLUT-1 and GLUT-3 receptors for glucose intake similarly to inflammatory cells; these receptors are known to be insensitive to insulin, fasting, and carbohydrate diet.^7^,^28^,^29^ By contrast, this sensitivity is very high for the GLUT-4 receptors, which are expressed by cardiomyocytes.^29^

The different patterns of cardiac ^18^F-FDG uptake documented herein at the 7th day of diet in both myocarditis and normal rats are best illustrated in the movies provided in a supplemental file and where the ring-like and myocarditis foci are shown to follow the left ventricular contraction motions.

It remains to be determined whether these results may be extrapolated to humans and also, whether such ketogenic diets might be even more effective and moreover, if they could be shortened when preceded and/or followed by short fasting periods, such as an overnight fast. A 7-day ketogenic diet is indeed too long to be routinely prescribed in all patients in this setting.

## New Knowledge Gained

One-week extension of a ketogenic diet:

1. Provides a gradual decrease in ^18^F-FDG uptake within normal myocardium of rats, reaching a lower level compared to a conventional 18-hour fasting protocol, but only at the 7th day of ketogenic diet.
2. Provides a high detectability of inflammatory areas by ^18^F-FDG-PET in rats.

## Conclusion

This experimental study shows that 1-week extension of a ketogenic diet provides a further decrease in the ^18^F-FDG uptake of normal myocardium and thus, a high detectability of inflammatory areas. Thereby, clinical trials, assessing prolonged ketogenic diets alone or in association with tolerable fasting periods, are warranted in this setting.

## Electronic supplementary material

Below is the link to the electronic supplementary material.

Supplementary file Cine-loop images recorded with ^18^F-FDG-TEP at the end of the 7 days of ketogenic diet in a myocarditis rat (A: vertical long-axis and B: horizontal long-axis) and in a normal rat (C: vertical long-axis and D: horizontal long-axis). The myocarditis area may be observed in the anterior wall of the myocarditis rat (white shadow in slice A) and areas of increased ^18^F-FDG uptake are observed in contact with the mitral annulus in all slices from both rats (dark shadows) (GIF 205 kb)

Supplementary material 2 (PPT 1505 kb)

## Abbreviations

^18^F-FDG: ^18^F-fluorodeoxyglucose
PET: Positron emission tomography
LV: Left ventricle
SD: Standard deviation
NS: No significant
HES: Hematoxylin eosin safan
M/B: Myocardium to blood ratio
