# Supplementary figures and images for: A 1-week extension of a ketogenic diet provides a further decrease in myocardial 18F-FDG uptake and a high detectability of myocarditis with FDG-PET
Source: J Nucl Cardiol. 2018 Aug 20;27(2):612–8. doi: 10.1007/s12350-018-1404-7 (PMC7174271; doi:10.1007/s12350-018-1404-7)

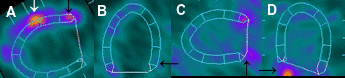

Supplement: Supplementary file 1 — Cine-loop images recorded with 18F-FDG-TEP at the end of the 7 days of ketogenic diet in a myocarditis rat (A: vertical long-axis and B: horizontal long-axis) and in a normal rat (C: vertical long-axis and D: horizontal long-axis). The myocarditis area may be observed in the anterior wall of the myocarditis rat (white shadow in slice A) and areas of increased 18F-FDG uptake are observed in contact with the mitral annulus in all slices from both rats (dark shadows) (GIF 205 kb) [file 12350_2018_1404_MOESM1_ESM.gif]
