# Supplementary material for: A 1-week extension of a ketogenic diet provides a further decrease in myocardial 18F-FDG uptake and a high detectability of myocarditis with FDG-PET
Source: J Nucl Cardiol. 2018 Aug 20;27(2):612–8. doi: 10.1007/s12350-018-1404-7 (PMC7174271; doi:10.1007/s12350-018-1404-7)
Supplement: Supplementary file 2 — Supplementary material 2 (PPT 1505 kb) [file 12350_2018_1404_MOESM2_ESM.ppt]

## Slide 1
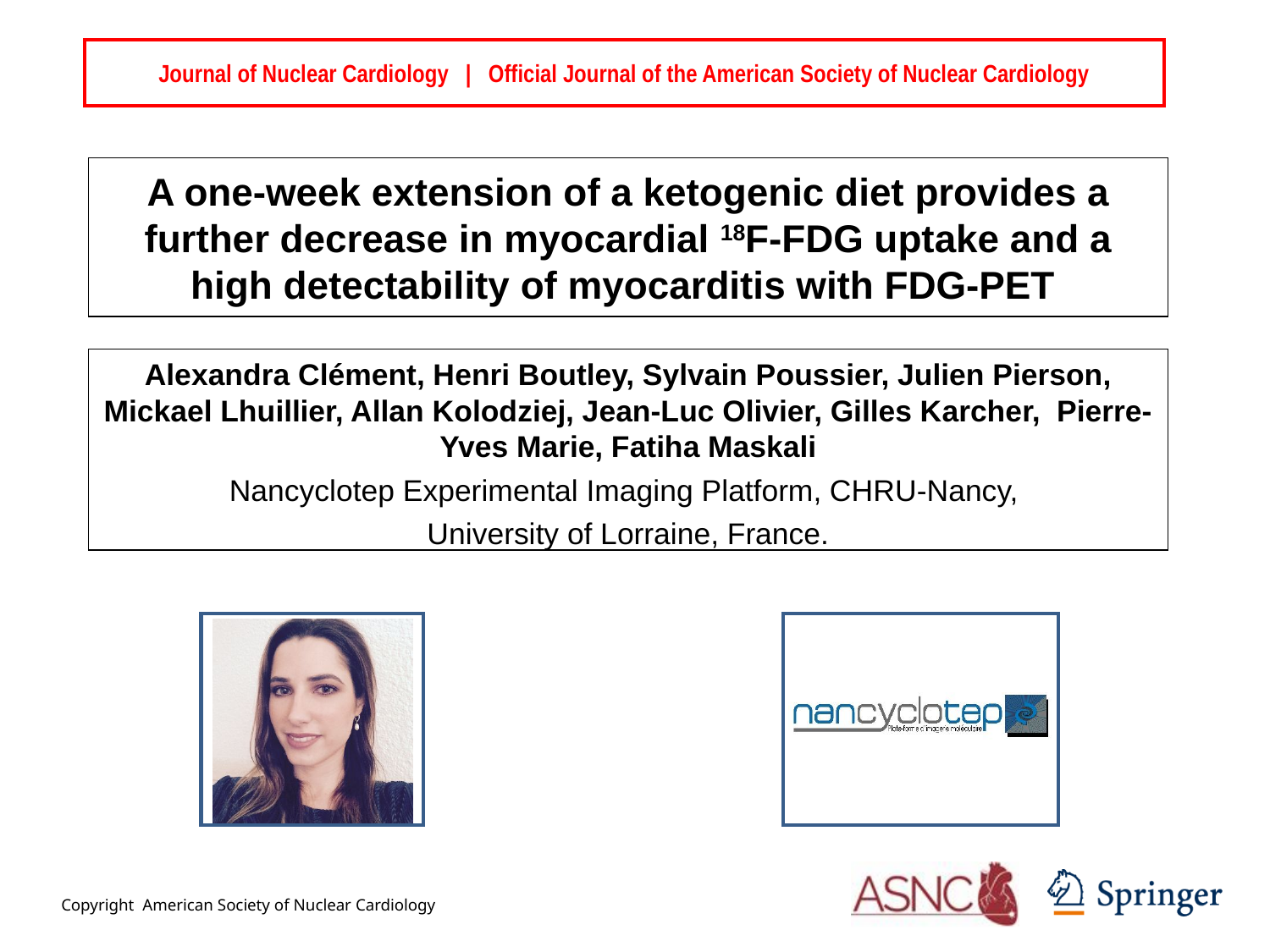

Journal of Nuclear Cardiology | Official Journal of the American Society of Nuclear Cardiology
# A one-week extension of a ketogenic diet provides a further decrease in myocardial 18F-FDG uptake and a high detectability of myocarditis with FDG-PET
Alexandra Clément, Henri Boutley, Sylvain Poussier, Julien Pierson, Mickael Lhuillier, Allan Kolodziej, Jean-Luc Olivier, Gilles Karcher, Pierre-Yves Marie, Fatiha Maskali
Nancyclotep Experimental Imaging Platform, CHRU-Nancy,
University of Lorraine, France.
Copyright American Society of Nuclear Cardiology

## Slide 2
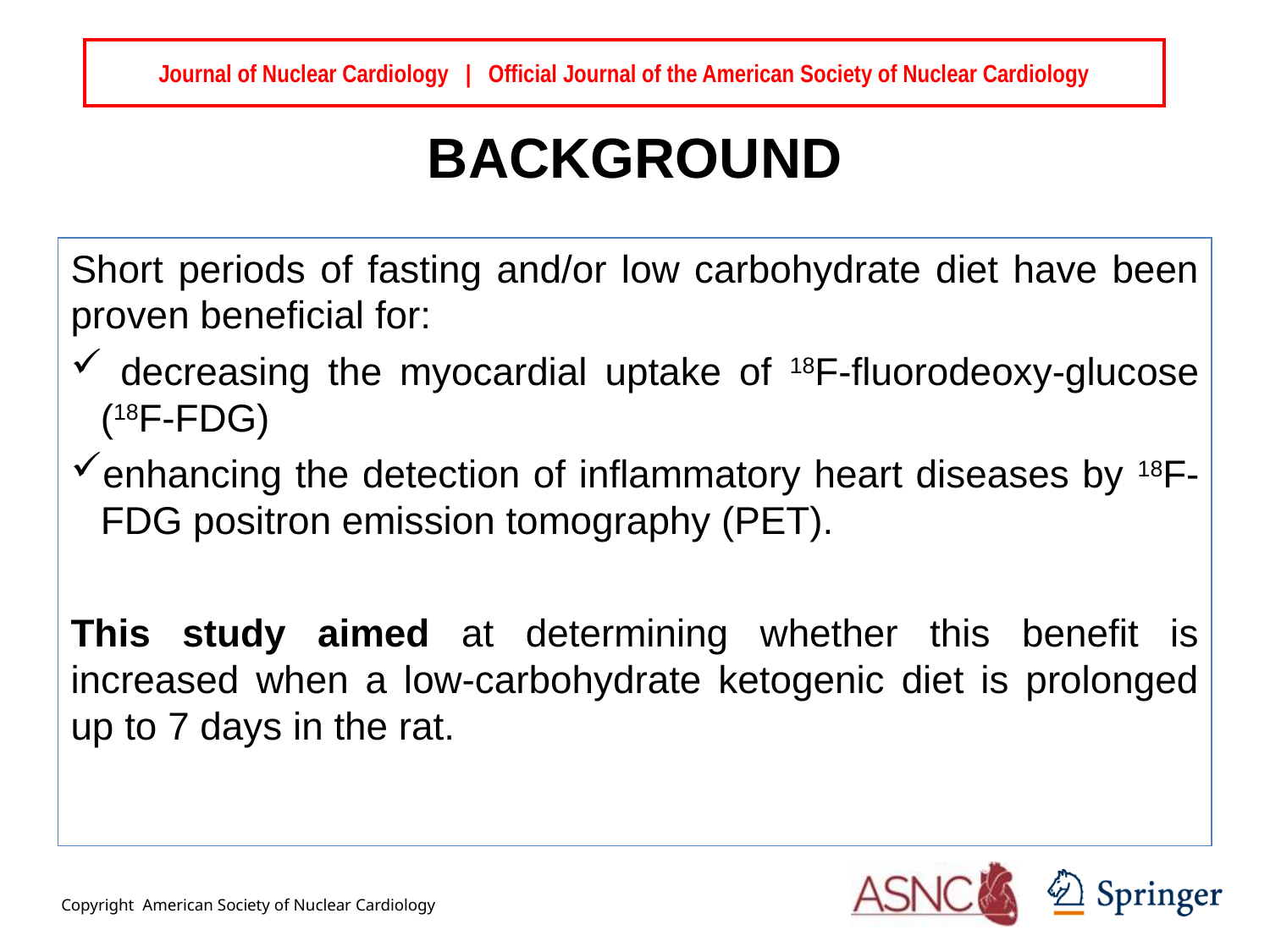

Journal of Nuclear Cardiology | Official Journal of the American Society of Nuclear Cardiology
# BACKGROUND
Short periods of fasting and/or low carbohydrate diet have been proven beneficial for:
 decreasing the myocardial uptake of 18F-fluorodeoxy-glucose (18F-FDG)
enhancing the detection of inflammatory heart diseases by 18F-FDG positron emission tomography (PET).
This study aimed at determining whether this benefit is increased when a low-carbohydrate ketogenic diet is prolonged up to 7 days in the rat.
Copyright American Society of Nuclear Cardiology

## Slide 3
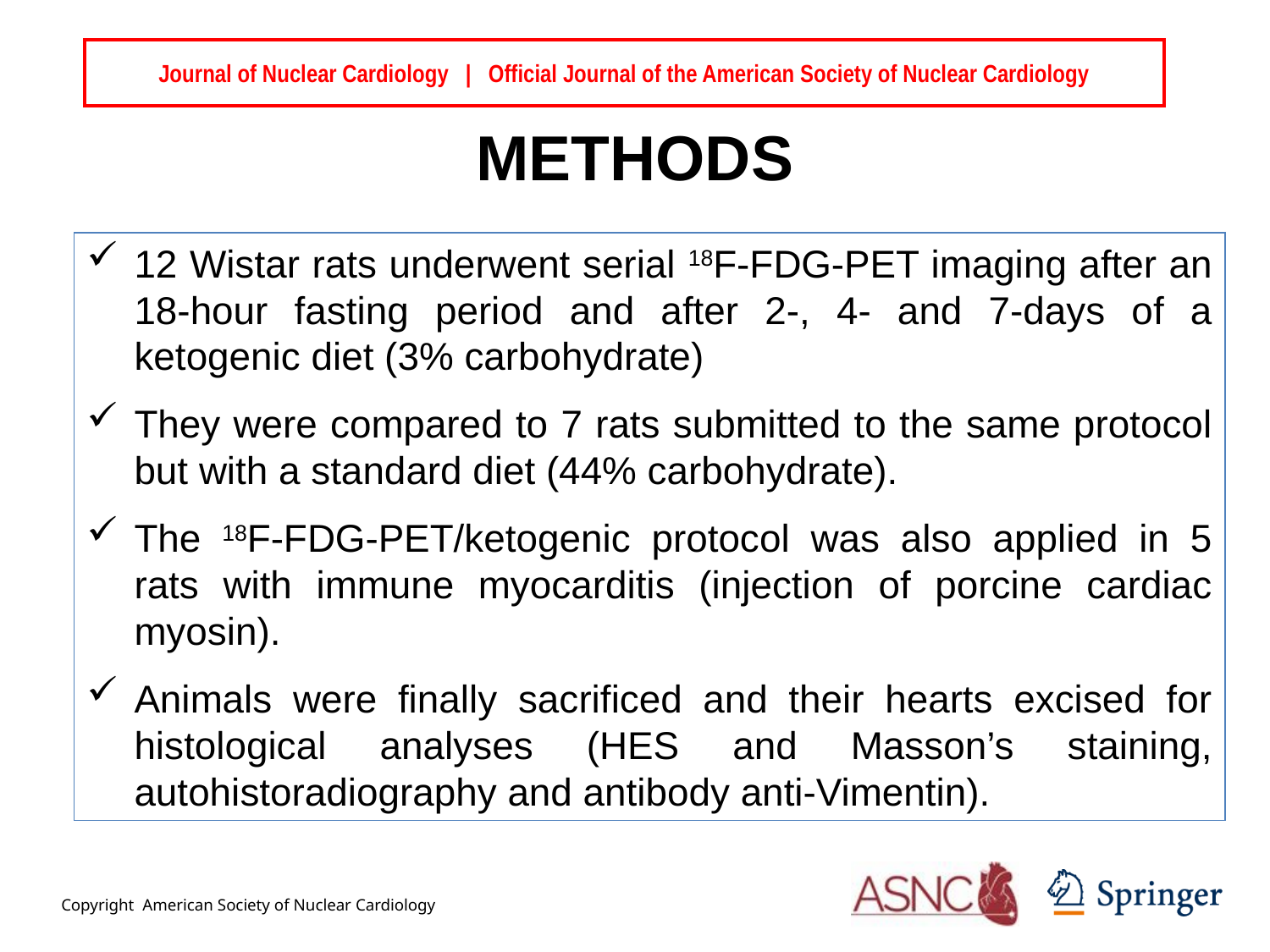

Journal of Nuclear Cardiology | Official Journal of the American Society of Nuclear Cardiology
# METHODS
12 Wistar rats underwent serial 18F-FDG-PET imaging after an 18-hour fasting period and after 2-, 4- and 7-days of a ketogenic diet (3% carbohydrate)
They were compared to 7 rats submitted to the same protocol but with a standard diet (44% carbohydrate).
The 18F-FDG-PET/ketogenic protocol was also applied in 5 rats with immune myocarditis (injection of porcine cardiac myosin).
Animals were finally sacrificed and their hearts excised for histological analyses (HES and Masson’s staining, autohistoradiography and antibody anti-Vimentin).
Copyright American Society of Nuclear Cardiology

## Slide 4
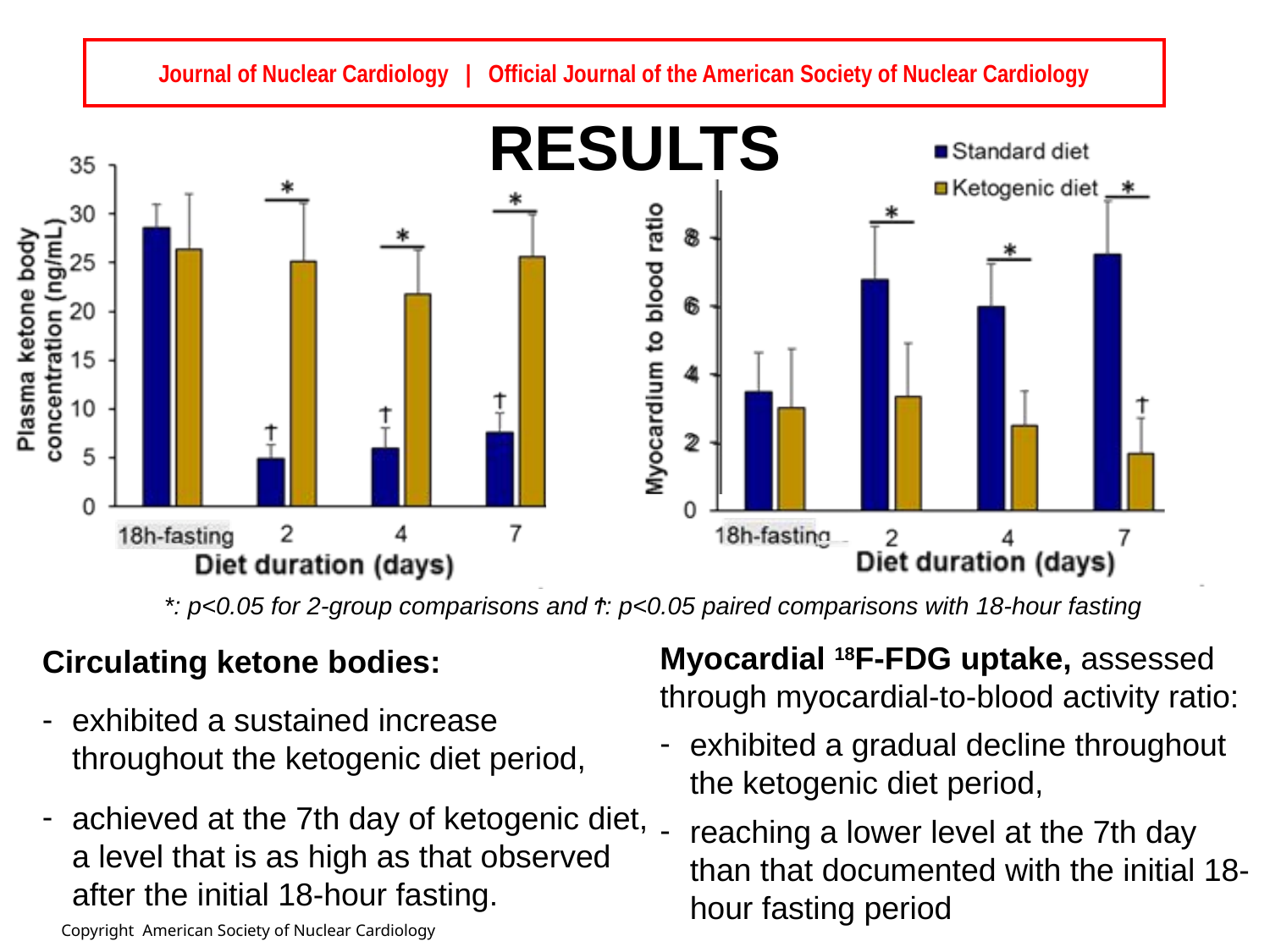

Journal of Nuclear Cardiology | Official Journal of the American Society of Nuclear Cardiology
# RESULTS
*: p<0.05 for 2-group comparisons and Ϯ: p<0.05 paired comparisons with 18-hour fasting
Myocardial 18F-FDG uptake, assessed through myocardial-to-blood activity ratio:
exhibited a gradual decline throughout the ketogenic diet period,
reaching a lower level at the 7th day than that documented with the initial 18-hour fasting period
Circulating ketone bodies:
exhibited a sustained increase throughout the ketogenic diet period,
achieved at the 7th day of ketogenic diet, a level that is as high as that observed after the initial 18-hour fasting.
Copyright American Society of Nuclear Cardiology

## Slide 5
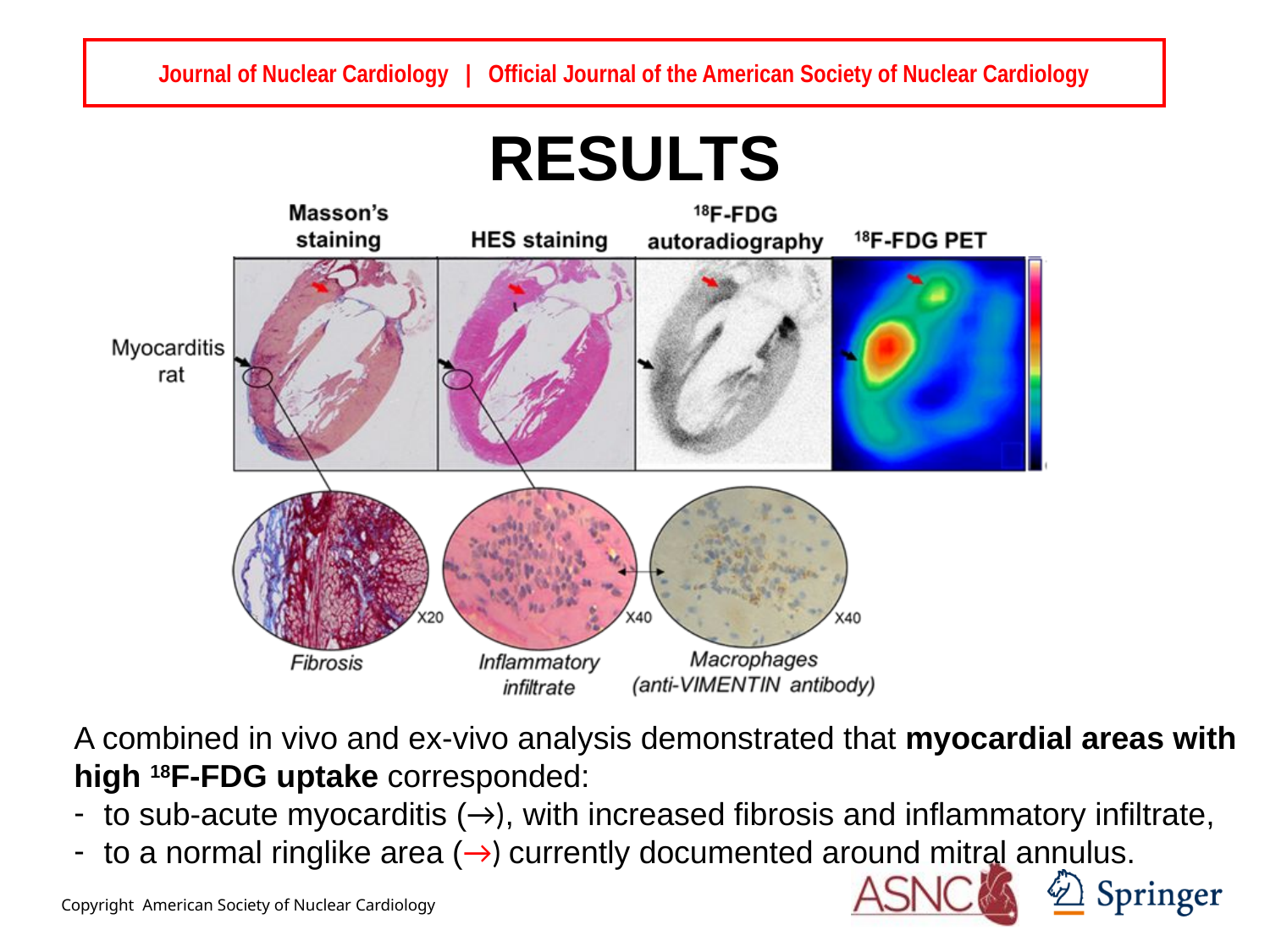

Journal of Nuclear Cardiology | Official Journal of the American Society of Nuclear Cardiology
# RESULTS
A combined in vivo and ex-vivo analysis demonstrated that myocardial areas with high 18F-FDG uptake corresponded:
to sub-acute myocarditis (→), with increased fibrosis and inflammatory infiltrate,
to a normal ringlike area (→) currently documented around mitral annulus.
Copyright American Society of Nuclear Cardiology

## Slide 6
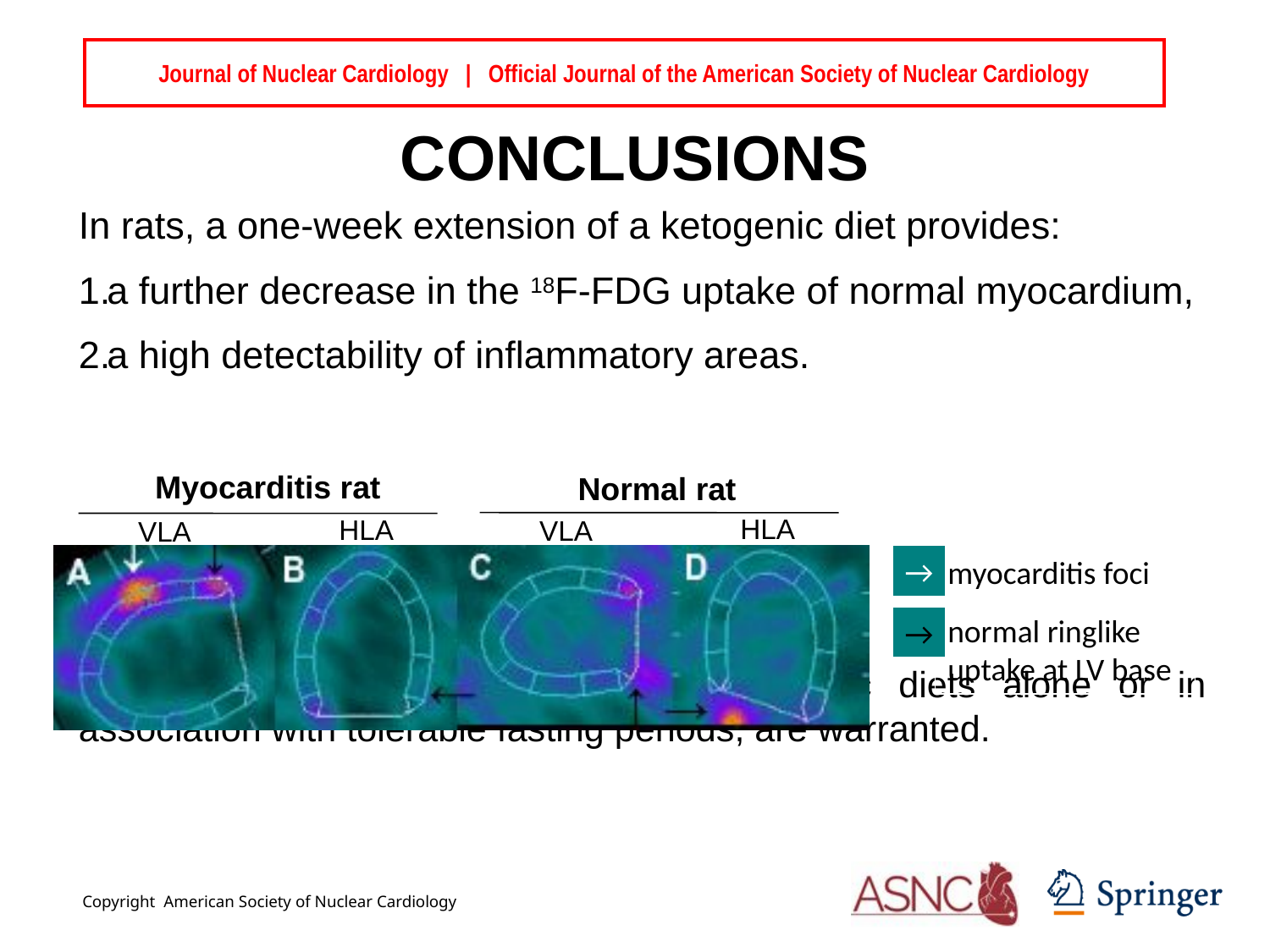

Journal of Nuclear Cardiology | Official Journal of the American Society of Nuclear Cardiology
# CONCLUSIONS
In rats, a one-week extension of a ketogenic diet provides:
a further decrease in the 18F-FDG uptake of normal myocardium,
a high detectability of inflammatory areas.
Clinical trials, assessing prolonged ketogenic diets alone or in association with tolerable fasting periods, are warranted.
Myocarditis rat
Normal rat
HLA
HLA
VLA
VLA
→
myocarditis foci
normal ringlike uptake at LV base
→
Copyright American Society of Nuclear Cardiology
